# Trends in psoriatic arthritis epidemiology in Poland

**DOI:** 10.1007/s00296-020-04734-x

**Published:** 2020-10-28

**Authors:** Małgorzata Tłustochowicz, Waldemar Wierzba, Michał Marczak, Witold Tłustochowicz, Andrzej Śliwczyński, Filip Raciborski, Brygida Kwiatkowska, Melania Brzozowska, Andrzej Jacyna, Bartłomiej Kisiel

**Affiliations:** 1 Department of Internal Diseases and Rheumatology, Military Institute of Medicine, Warsaw, Poland; 2 Satellite Campus in Warsaw, University of Humanities and Economics in Lodz, Łódź, Poland; 3 Department of Management and Logistics in Health Care, Medical University of Lodz, Łódź, Poland; 4 Department of Prevention of Environmental Hazards and Allergology, Medical University of Warsaw, Warsaw, Poland; 5 Early Arthritis Clinic, National Institute of Geriatrics, Rheumatology and Rehabilitation, Warsaw, Poland; 6 Department of Drug Management, National Health Fund, Warsaw, Poland; 7 National Health Fund, Warsaw, Poland

**Keywords:** Psoriasis, Psoriatic arthritis, Prevalence, Epidemiology

## Abstract

Psoriatic arthritis (PsA) is a heterogeneous inflammatory arthritis, usually seronegative and associated with psoriasis (Ps). The prevalence and incidence of psoriatic arthritis show strong ethnic and geographic variations. The aim of the study was to assess the epidemiological trends in psoriatic arthritis in Poland. The National Health Fund (NHF) database for the period 2008–2018 was analyzed. PsA was defined as ICD-10 codes L40.5, M07, M07.0, M07.1, M07.2 and M07.3, while psoriasis as ICD-10 codes L40 and L40.X (L40.0 to L40.9). A steady increase in the number of PsA patients (from 16,790 to 32,644) and in PsA recorded prevalence (from 38.47 per 100,000 in 2008 to 73.11 per 100,000 in 2018) was observed between 2008 and 2018. The PsA/Ps ratio increased to a similar extent (from 8.3 to 17.5%). The percentage of PsA patients receiving rehabilitation services remained constant throughout the observation period (mean: 17.35%; range 16.7–18.9%). The study showed a steady and continuous increase in PsA recorded prevalence. A simultaneous increase in the PsA/Ps ratio suggests that the main reason for the observed trend is greater disease detection .

## Introduction

Inflammatory arthritis is an important cause of morbidity and disability in industrialized countries. The current data show that the incidence and prevalence of polyarthritis in Poland are as high as 284.7 per 100,000 and 2030.1 per 100,000, respectively [1]. Psoriatic arthritis (PsA) is one of the most common form of polyarthritis. It is a chronic inflammatory disease involving the articular and periarticular structures, usually seronegative and most often associated with psoriasis [2]. According to the current classification PsA belongs to the group of spondyloarthritis. Initially, PsA was considered a relatively benign disorder; however, a growing body of evidence shows that PsA is a destructive and progressive disease, with a similar impact on quality of life and functional status as rheumatoid arthritis [3]. Moreover, it was also found that PsA associates with several comorbidities, including cardiovascular disease and results in increased mortality [4–7].

Although the prevalence and incidence of PsA show strong ethnic and geographic variations [8] substantial differences are also seen between studies conducted in same populations [8–10]. This large heterogeneity of epidemiological data is due to several factors, including lack of uniform diagnostic criteria, different study period (several studies show substantial temporal changes in PsA epidemiology), different study design (population-based studies or hospital/clinic-based reports; general population or specific groups, e.g. adults etc.) and different case definitions (e.g. self-reporting, expert’s diagnosis, analysis of healthcare system databases etc.).

In 2016, our group published a study on the PsA epidemiology in Poland [11]. The study involved the analysis of National Health Fund (NHF) database and PsA cases were defined by ICD-10 codes M07 and L40.5. It was a whole-population study, as NHF is the only one entity providing public health insurance in Poland and its database contains data of all health services (including ICD-10 codes) financed from public funds. Strikingly, we found a relatively low prevalence of PsA in Polish population—3.2 per 10,000 (data for 2015) as compared to 13.3 per 10,000 in a recent meta-analysis by Scotti et al. [10]. The previous study involved exclusively patients with ICD-10 codes specific for PsA (M07.0, M07.1, M07.2, M07.3 and L40.5). As using four-character codes (i.e. M07.X) is not mandatory in health services reports, we may suspect that some PsA patients were coded with a three-character code (i.e. M07). This, in turn, might have led to substantial underestimation of PsA prevalence in our previous study. Thus, we decided to reassess the NHF data with the extension of the analysis to the year 2018. Beyond evaluating PsA prevalence in the general population we aimed to assess its prevalence in Ps patients and to evaluate the percentage of PsA patients receiving rehabilitation.

## Materials and methods

We analyzed NHF database to assess temporal trends in: (i) total and regional PsA recorded prevalence in general population, (ii) PsA recorded prevalence among psoriasis patients and (iii) amount of rehabilitation services provided to PsA patients.

SQL (Structured Query Language) tools were used to extract the data (using a filter in accordance with the assumed scope of ICD-10 diagnosis). To comply with the principles of processing sensitive data [12], such as personal ID (PESEL), data were depersonalized before the analysis, i.e. the patients’ data devoid of sensitive data were processed and analyzed. PsA was defined as ICD-10 codes L40.5, M07, M07.0, M07.1, M07.2 and M07.3, while psoriasis as ICD-10 codes L40 and L40.X (L40.0 to L40.9).

The statistical analyses were performed with the use of SAS EG 7.1 software. The prevalence rates were calculated per 100,000 inhabitants, based on demographic data obtained from the Statistics Poland website (https://stat.gov.pl/en/) for each year separately (state as of 31 December). The mean values are given with standard deviations (age of patients) or ranges (prevalence, percentage of cases).

It should be emphasized that the definition of Ps and PsA used in current study may lead to a certain overestimation of prevalence as patients with a suspicion of Ps/PsA may also be reported with the above ICD-10 codes. We may assume that such situation may be relatively common in general outpatient health services (as compared to specialist outpatient health services and hospitalizations). Therefore, although patients reported to NHF by family physicians are included in the total number of PsA patients (Table 1), they were not included in the calculations of prevalence (in these analyses only patients reported within specialist outpatient clinics and hospitalized patients were included).

**Table 1:** The number of PsA patients (ICD-10 codes M07, M07.0, M07.1, M07.2, M07.3, L40.5) in subsequent years in Poland

	2008	2009	2010	2011	2012	2013	2014	2015	2016	2017	2018
Total	16,851	19,624	20,063	21,452	23,006	24,521	25,997	27,800	29,771	31,313	32,644
SOC	13,656	14,926	15,375	16,506	17,877	19,064	20,314	21,338	22,599	23,513	24,491
HOS	1872	2651	3013	3288	3580	3944	3898	4412	4682	5034	5224
SOC + HOS	14,460	16,193	16,816	18,063	19,425	20,880	22,046	23,375	24,852	26,112	27,208
Men (SOC + HOS)	5157	5990	6329	6702	7224	7802	8183	8703	9166	9718	10,031
M07 + M07.X (SOC + HOS)	13,706	14,673	15,008	15,922	17,054	17,939	19,024	19,921	20,818	21,146	21,666
L40.5 (SOC + HOS)	1510	2894	3334	3866	4445	5057	5213	5820	6444	7253	7918

Total—patients reported by general and specialist outpatient clinics and hospitalized patients; *SOC* specialist outpatient clinics; *HOS* hospitalizations; *M07.X* patients with codes M07.0, M07.1, M07.2 and M07.3

To assess the probable structure of the group of patients reported to the NHF with the three-character code M07 we compared the number of patients with PsA-specific M07.X codes (i.e. M07.0, M07.1, M07.2 and M07.3) and inflammatory bowel disease (IBD)-specific M07.X codes (i.e. M07.4, M07.5, M07.6) in years 2008–2018 and found that the number of PsA-specific codes was on average 13.18 times higher (range 11.13–16.09) as compared to IBD-specific codes. Therefore, it should be assumed that: (i) the vast majority of patients reported using the M07 code are patients with PsA; (ii) the method used in our previous study [11] caused a significant underestimation of PsA prevalence; (iii) inclusion of subjects with M07 code should not lead to a significant overestimation of PsA prevalence (vast majority of those subjects do have PsA).

Given the fact that the study was based on a retrospective analysis of NHF database, the patients’ written informed consent was not required and ethical approval was not needed.

## Results

The number of PsA patients reported in particular years is summarized in Table 1. From 2008 to 2018 there has been a steady increase in the number of PsA patients: from 16,790 to 32,644 if reports from general outpatient clinics (GOC) were included and from 14,460 to 27,208 if only reports from specialist outpatient clinics (SOC) and hospitalizations (HOS) were taken into account. Men accounted for 36–37% of all PsA patients in individual years. The mean age was slightly higher in female PsA patients (54.93 ± 13.18 years vs 51.91 ± 13.28 years).

**Fig. 1:**
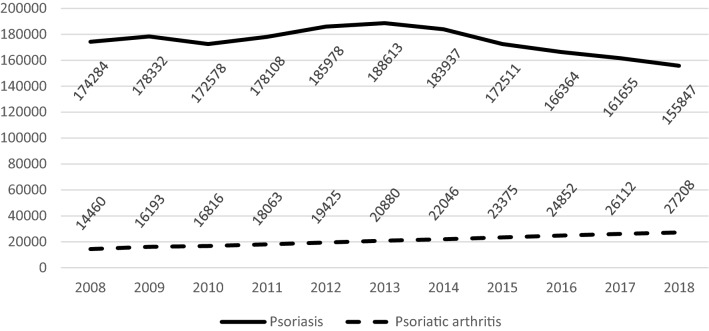
The number of Ps and PsA patients reported by SOC and hospitalized in subsequent years in Poland

The number of PsA patients in particular voivodeships (regions) is presented in Table 2 (only data from SOC and HOS are included). The highest numbers of patients with a diagnosis of PsA were reported in Mazowieckie and Śląskie voivodeships, the lowest—in Lubuskie, Opolskie and Warmińsko-Mazurskie. The PsA prevalence in Poland and in particular voivodeships in subsequent years is presented in Table 3. A steady increase of prevalence was observed in the whole country (from 38.47 per 100,000 in 2008 to 73.11 per 100,000 in 2018) and in all its regions. The highest prevalence was observed for Kujawsko-Pomorskie and Pomorskie voivodeships (in 2018: 95.05 per 100,000 and 85.56 per 100,000, respectively), the lowest—in Lubuskie, Łódzkie and Warmińsko-Mazurskie voivodeships (in 2018: 48.06 per 100,000; 50.64 per 100,000 and 53.69 per 100,000, respectively). The percentage of PsA patients receiving rehabilitation services remained constant throughout the observation period (mean: 17.35%; range: 16.7-18.9%). The prevalence of psoriasis in years 2008–2018 ranged from 0.42 to 0.51% with a mean value of 0.47%; a slight decrease in the prevalence was observed after year 2013 (0.51% in 2013 as compared to 0.42% in 2018). The increase of PsA prevalence in combination with the slight decrease in Ps prevalence resulted in gradual increase of PsA/Ps ratio (Fig. 1)—from 8.3% in 2008 to 17.5% in 2018.

**Table 2:** The number of PsA patients (ICD-10 codes M07, M07.0, M07.1, M07.2, M07.3, L40.5) reported by SOC and hospitalized (i.e. patients reported by GOC are not included) in subsequent years in particular voivodeships

	2008	2009	2010	2011	2012	2013	2014	2015	2016	2017	2018
01	991	1211	1282	1336	1428	1505	1583	1665	1726	1807	1872
02	1053	1064	1184	1369	1625	1607	1702	1817	1915	1941	1977
03	730	840	798	836	908	962	1131	1210	1240	1321	1395
04	228	300	376	430	502	456	458	396	494	474	488
05	574	734	712	826	838	877	953	1024	1074	1145	1251
06	1017	1237	1270	1454	1694	1852	2009	2220	2339	2527	2740
07	1613	1964	2026	2267	2885	2844	3053	3291	3911	4035	4140
08	436	516	563	536	530	537	596	612	655	722	722
09	884	969	987	1206	1193	1351	1389	1517	1531	1559	1740
10	365	380	438	498	520	535	609	629	688	812	853
11	1389	1539	1595	1528	1676	1778	1625	1676	1705	1875	1992
12	2326	2447	2520	2527	2696	2905	2990	3100	3257	3294	3439
13	412	443	414	484	528	607	639	649	704	769	831
14	480	473	472	507	472	539	630	644	674	747	767
15	1210	1306	1373	1465	1529	1802	1898	2023	2150	2266	2266
16	830	878	914	916	1006	1068	1160	1258	1285	1274	1258

*GOC* general outpatient clinics, *SOC* specialist outpatient clinics, *01* Dolnośląskie, *02* Kujawsko-Pomorskie, *03* Lubelskie, *04* Lubuskie, *05* Łódzkie, *06* Małopolskie, *07* Mazowieckie, *08* Opolskie, *09* Podkarpackie, *10* Podlaskie, *11* Pomorskie, *12* Śląskie, *13* Świętokrzyskie, *14* Warmińsko-Mazurskie, *15* Wielkopolskie, *16* Zachodniopomorskie

**Table 3:** PsA prevalence per 100,000 inhabitants (based on SOC reports and HOS) in subsequent years in particular voivodeships

	2008	2009	2010	2011	2012	2013	2014	2015	2016	2017	2018
01	34.44	42.10	43.96	45.82	48.98	51.70	54.43	57.31	59.44	62.26	64.53
02	50.92	51.42	56.41	65.25	77.46	76.74	81.40	87.03	91.87	93.19	95.05
03	33.77	38.94	36.58	38.44	41.87	44.53	52.56	56.46	58.06	62.04	65.75
04	22.60	29.70	36.76	42.03	49.07	44.61	44.87	38.84	48.55	46.62	48.06
05	22.52	28.88	27.96	32.55	33.14	34.83	37.99	40.99	43.16	46.17	50.64
06	30.94	37.50	38.13	43.52	50.56	55.17	59.72	65.86	69.28	74.63	80.69
07	30.99	37.61	38.54	42.98	54.50	53.59	57.34	61.62	73.01	75.10	76.78
08	42.21	50.04	55.27	52.78	52.36	53.33	59.45	61.27	65.86	72.84	73.08
09	42.11	46.10	46.40	56.68	56.04	63.47	65.26	71.31	71.99	73.27	81.74
10	30.63	31.94	36.37	41.43	43.34	44.71	51.03	52.85	57.93	68.51	72.12
11	62.58	69.01	70.24	67.04	73.29	77.56	70.69	72.72	73.76	80.83	85.56
12	50.07	52.73	54.34	54.58	58.35	63.06	65.09	67.72	71.36	72.35	75.75
13	32.37	34.88	32.22	37.81	41.40	47.79	50.50	51.51	56.12	61.53	66.78
14	33.64	33.14	32.46	34.89	32.51	37.22	43.58	44.65	46.88	52.06	53.59
15	35.61	38.32	39.90	42.46	44.21	52.03	54.71	58.23	61.82	65.02	64.92
16	49.03	51.85	53.01	53.16	58.42	62.11	67.52	73.40	75.20	74.65	73.87
POLAND	**38.47**	**43.06**	**44.35**	**47.75**	**52.71**	**55.91**	**59.24**	**62.64**	**66.94**	**70.01**	**73.11**

*SOC* specialist outpatient clinics, *HOS* hospitalizations, *01* Dolnośląskie, *02* Kujawsko-Pomorskie, *03* Lubelskie, 04 Lubuskie, *05* Łódzkie, *06* Małopolskie, *07* Mazowieckie, *08* Opolskie, *09* Podkarpackie, *10* Podlaskie, *11* Pomorskie, *12* Śląskie, *13* Świętokrzyskie, *14* Warmińsko-Mazurskie, *15* Wielkopolskie, *16* Zachodniopomorskie

## Discussion

The actual PsA prevalence is difficult to estimate as the results of epidemiological studies vary widely. This heterogeneity is due to number of factors, e.g. differences in methodology, target populations and criteria used to define PsA. A recent meta-analysis by Scotti et al. reported the random effect pooled PsA prevalence at 133 per 100,000 [10] but the between-study heterogeneity was extremely high (*I*^*2*^ index 99.3%).

In 2016 we published a study on the PsA epidemiology in Poland [11] reporting its prevalence to be at 32 per 100,000 (in 2015). Currently presented results indicate that the prevalence reported in the previous study was clearly underestimated, and actual epidemiological indicators are almost twice as high. In 2018 the Ps and PsA recorded prevalence in Poland was at 0.42% and 0.073%, respectively, with a PsA:Ps ratio of 0.175. As compared to 2008 the PsA recorded prevalence doubled, while the Ps recorded prevalence decreased slightly from 0.46% to 0.42%.

The reported PsA prevalence is lower as compared to the meta-analysis by Scotti et al. [10]. This discrepancy may be due to few factors. First, prevalence estimates in Europe vary significantly, from 0.05% in Turkey and the Czech Republic to 0.21% in Sweden [13]. Second, the estimates of prevalence depend highly on the method of PsA cases identification; e.g. prevalence estimates in the United States range from 0.06 to 0.25% with the lowest estimate derived from a study utilizing ICD codes and the highest from articles using patient self-report of PsA diagnosis [13]. The meta-analysis by Scotti et al. included studies utilizing various method of PsA cases identification. Third, the PsA prevalence increases with age with a peak > 50 years [9], therefore, it will higher in studies involving only adults compared to studies covering the entire population; we estimate that if the study was limited to adults only, the prevalence of the disease would increase by 15–20% (as the percentage of people aged 0–17 in Poland is about 18%). Fourth, our study may slightly underestimate the PsA prevalence, as cases reported exclusively by general practitioners were not included in the calculation of prevalence—the inclusion of those cases would increase PsA prevalence by 20% to 0.088%. Thus, it can be estimated that taking into account both of the above factors (i.e. including data from family doctor clinics and limiting the study to adults) could potentially increase the PsA prevalence by 35–40% to around 0.1%.

A similar methodology to ours (the use of diagnostic code to identify Ps and PsA patients) was used in a population-based study by Eder et al. [14] performed in Ontario, Canada. This study yielded Ps and PsA prevalence of 2.54% and 0.17%—much higher than in our study. This discrepancy may be partly due to ethnic differences, however, the fact that the study covered only people over 20 years old was undoubtedly also significant.

The average prevalence of psoriasis in European and North American populations is estimated at 2-3%, with significant differences between countries. The Ps prevalence in population-based studies from Europe ranges from 1.3 to 8.5% [15]. The prevalence of ~ 0.5%, found in our study, is therefore markedly lower. As in the case of PsA, this difference may be due to a number of factors. On one hand, our study was able to capture only patients using health services. As psoriasis is a disease with exacerbation and remission, and in some cases its course is mild (these patients may relatively rarely use health services), the Ps prevalence found in our study may be lower than the actual. On the other hand, this relatively low prevalence may be partly due to the fact that all age groups were included in the study, and psoriasis is relatively rare among children, especially the youngest. In this context, it is worth mentioning a study by Lӧfvendahl et al. [16]. The target population included all residents (also children) in the Skåne region in southern Sweden and the identification of Ps and PsA cases was based on ICD-10 codes. The study yielded a Ps prevalence of 1.53%—lower than the average prevalence of 2–3% (despite the fact that the study concerned Northern European population in which we would expect a relatively high disease prevalence).

Our study showed a steady increase of PsA between 2008 and 2018. This finding is not surprising as previous studies showed similar results [14, 17]. This time trend may be related to a true increase in disease expression. However, a more plausible explanation is the greater detectability of the disease resulting from an increasing awareness among physicians to detect PsA as early as possible and more sensitive diagnostic methods. The latter possibility is further confirmed by the fact that we have not found a parallel increase in the prevalence of psoriasis—Ps showed a slightly decreasing prevalence between 2008 and 2018. These results are in contrast to those by Eder et al. [14] who observed a 45% increase of prevalence between 2000 and 2015. Different results may be partly related to ethnic differences and different period under analysis, but the opposite epidemic trend is surprising; it is possible that in case of Poland socio-economic factors related to the opening of the European Union labor market for Polish workers may have influenced the results; in recent years, even several million Poles have temporarily worked abroad—they still were “visible” in the calculations of the Statistics Poland, but, as they did not use healthcare in Poland, they could not be “captured” by our analysis based on the National Health Fund data. If this is the case, the actual PsA prevalence may also be higher than observed in current study.

Our study showed a rising PsA/Ps ratio—it increased from 0.083 in 2008 to 0.175 in 2018. As mentioned above, these results suggest an improving detection of PsA. Prevalence of PsA has been reported to be in a wide range among patients with psoriasis (5.8–40.9%) [9]. A recent systematic review and meta-analysis by Alinaghi et al. found a pooled PsA prevalence of 22.7% for Europe [18]. This figure is close to ours, suggesting that PsA detectability in Poland is at the similar level as compared to other European countries. However, it appears that many PsA cases remain undiagnosed. According to a meta-analysis by Villani et al. the prevalence of undiagnosed PsA among Ps patients is 15.5% [19]. Similar conclusions can be drawn from the study of Maese et al. [20] in which PsA was diagnosed in 285 of 949 consecutive psoriatic patients attending dermatology centers in 7 European and North American countries; of these 285 patients 117 had not been given the diagnosis of PsA before study entry. Moreover, the study highlighted the key role of the clinical examination by rheumatologist in the diagnosis of PsA (imaging and laboratory tests had a marginal effect on the detection of PsA). The results of the above-mentioned studies may suggest that in Poland up to 40-50% of PsA patients remain undiagnosed. Diagnostic delays may in turn lead to serious health consequences—a study by Harron et al. showed that the diagnostic delay of even 6 months can lead to significantly more radiographic damage and worse physical function [21]. In order to ensure early detection of the disease, it seems crucial to promptly refer a patient with a suspected PsA to a rheumatologist as distinguishing PsA from other joint diseases may be challenging for a non-rheumatologist [22].

The percentage of PsA patients receiving rehabilitation services was relatively low and remained stable during the entire period under estimation. This finding indicates that rehabilitation is relatively rarely used in the treatment of PsA. It is worth mentioning here that the GRAPPA recommendations include physiotherapy as an element of PsA therapy, especially axial form and possibly enthesitis [23].

The main strength of our study is the use of NHF data—a centralized data resource of a public health system. As the NHF is the only provider of public health insurance in Poland, the study had a potential to capture virtually all Ps and PsA patients in Poland.

We acknowledge several limitations of the study. The study based on the analysis of medical codes does not allow distinguishing patients with PsA from subjects with suspected PsA, which can lead to some overestimation of the prevalence. On the other hand, the study was unable to capture patients not using healthcare system or using exclusively private healthcare system (although the percentage of people using only private health care is low in Poland) which might have resulted in a certain underestimation of prevalence. It should also be mentioned that similarly to our previous study [11] subjects with code M09.0 were not included in the analysis; however, this had practically no effect on the results of the study as the average total number of M09 cases (i.e. M09 and M09.X codes) was 80 in years 2008–2016 (i.e. ~ 0.3% of all cases).

In summary, we observed a steady increase in PsA prevalence between 2008 and 2018—from 38.47 per 100,000 to 73.11 per 100,000. A similar increase was found for PsA/Ps ratio (from 8.3% to 17.5%). Although the results of our study indicate an improving detection of PsA, still many cases of the disease (possibly up to 50%) remain undiagnosed. This points to the need of development of the new diagnostic tools and criteria on the one hand and of increasing the awareness of PsA among patients and physicians (especially general practitioners) on the other hand.
